# Multi-Spectroscopic Characterization of Human Serum Albumin Binding with Cyclobenzaprine Hydrochloride: Insights from Biophysical and *In Silico* Approaches

**DOI:** 10.3390/ijms20030662

**Published:** 2019-02-03

**Authors:** Mohammad Hassan Baig, Safikur Rahman, Gulam Rabbani, Mohd Imran, Khurshid Ahmad, Inho Choi

**Affiliations:** 1 Department of Medical Biotechnology, Yeungnam University, 280 Daehak-ro, Gyeongsan, Gyeongbuk 38541, Korea; mohdhassanbaig@gmail.com (M.H.B.); shafique2@gmail.com (S.R.); rbbgulam@gmail.com (G.R.); ahmadkhursheed2008@gmail.com (K.A.); 2 Department of Biophysics, All India Institute of Medical Sciences, Ansari nagar, New Delhi 110029, India; imranimran.amu@gmail.com

**Keywords:** muscle relaxant, circular dichroism, cyclobenzaprine hydrochloride, esterase-like activity, human serum albumin, molecular docking, molecular dynamics

## Abstract

Cyclobenzaprine hydrochloride (CBH) is a well-known muscle relaxant that is widely used to relieve muscle spasms and other pain associated with acute musculoskeletal conditions. In this study, we elucidated the binding characteristics of this muscle relaxant to human serum albumin (HSA). From a pharmaceutical and biochemical viewpoint, insight into the structure, functions, dynamics, and features of HSA-CBH complex holds great importance. The binding of CBH with this major circulatory transport protein was studied using a combination of biophysical approaches such as UV-VIS absorption, fluorescence quenching, and circular dichroism (CD) spectroscopy. Various *in silico* techniques, molecular docking and molecular dynamics, were also used to gain deeper insight into the binding. A reduction in the fluorescence intensities of HSA-CBH complex with a constant increase in temperature, revealed the static mode of protein fluorescence quenching upon CBH addition, which confirmed the formation of the HSA-CBH ground state complex. The alteration in the UV-VIS and far-UV CD spectrum indicated changes in both secondary and tertiary structures of HSA upon binding of CBH, further proving CBH binding to HSA. The analysis of thermodynamic parameters ∆H° and ∆S° showed that binding of CBH to HSA was dominated by intermolecular hydrophobic forces. The results of the molecular docking and molecular dynamics simulation studies also confirmed the stability of the complex and supported the experimental results.

## 1. Introduction

**Figure 1:**
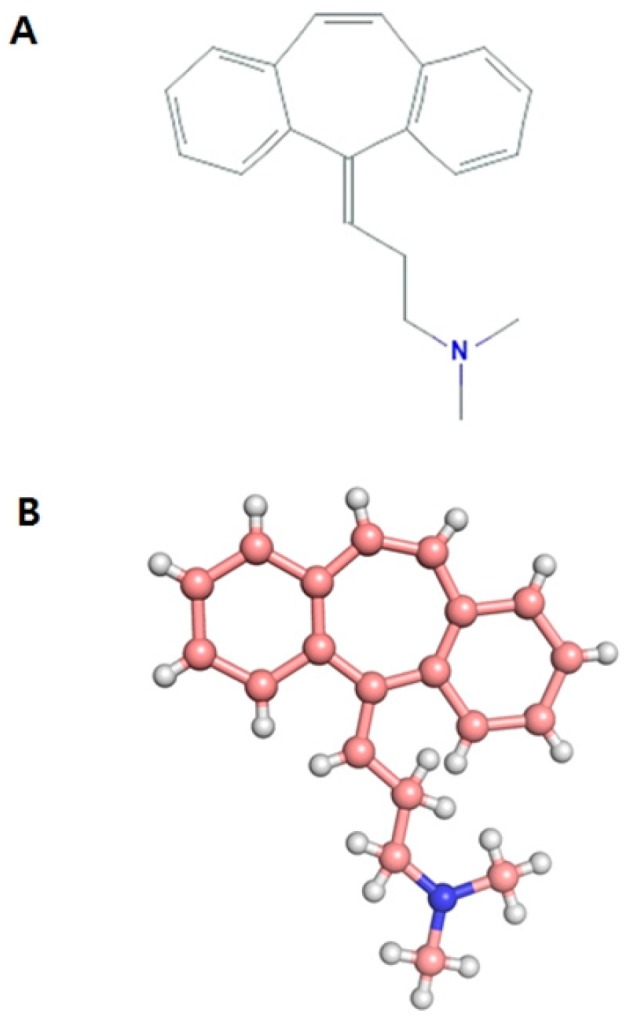
Structural representations of cyclobenzaprine: (**A**) 2-D structure and (**B**) 3-D structure.

Skeletal muscle, the largest organ of the human body, comprises 30–40% of the total body weight [1]. In vertebrates, skeletal muscles provide locomotive ability and plays important metabolic and endocrine roles in the organism [2,3]. Skeletal muscle relaxants are a diverse group of medications used to treat muscle spasms and pain. Cyclobenzaprine hydrochloride (CBH) (Figure 1), a 5-HT2 receptor antagonist, is a well-known muscle relaxant. It is widely used to relieve muscle spasms and other pain associated with acute musculoskeletal conditions [4,5] and is also reported to have antidepressant activity [6].

Human serum albumin (HSA) is the most abundant plasma protein and is found in nearly all mammals [7,8]. HSA, a 585 amino acid protein, is mainly synthesized in the liver and is secreted into the intravascular space [9,10]. It is a globular α-helical protein classified into three different functional domains: I, II and III [11]. The structure of HSA shows that domain I is composed of amino acids 1–195, while domain II is composed of amino acids 196–383 and domain III consists of amino acids 384–585 [12].

HSA serves as a carrier protein for a large number of drugs and bioactive molecules [13]. In humans, most drugs circulate into the blood by reversibly binding to HSA [14]. HSA has one Trp214 in subdomain IIA that is the major intrinsic fluorophore and is highly sensitive to micro-environmental changes. In-depth knowledge of the interaction between HSA and a drug is extremely important for understanding the pharmacokinetics and pharmacodynamics of a drug [15,16]. Therefore, studies focusing on the interaction of a drug with HSA are significant in clinical medicine, life sciences and chemistry [10,17]. Such studies are very helpful in determining the various properties of a drug, such as its distribution, secretion, metabolism, etc. [18]. Previously we thoroughly investigated the interaction of HSA with two different muscle relaxants, eperisone hydrochloride (EH) and tolperisone hydrochloride [8,10].

In this study, our aim was to investigate the binding mechanism of CBH with HSA and to gain deeper insight into the interaction mechanism. The combination of UV-VIS [19], fluorescence [20,21] and circular dichroism (CD) [22,23] spectroscopy, along with *in silico* approaches, thoroughly describe the structural alterations within HSA after binding to CBH. Further valuable information about the binding mode, binding mechanism, binding constants, and structural changes within the HSA-CBH complex are also widely explored.

## 2. Results and Discussion

### 2.1. UV-Visible Spectroscopy of HSA-CBH Complex

**Figure 2:**
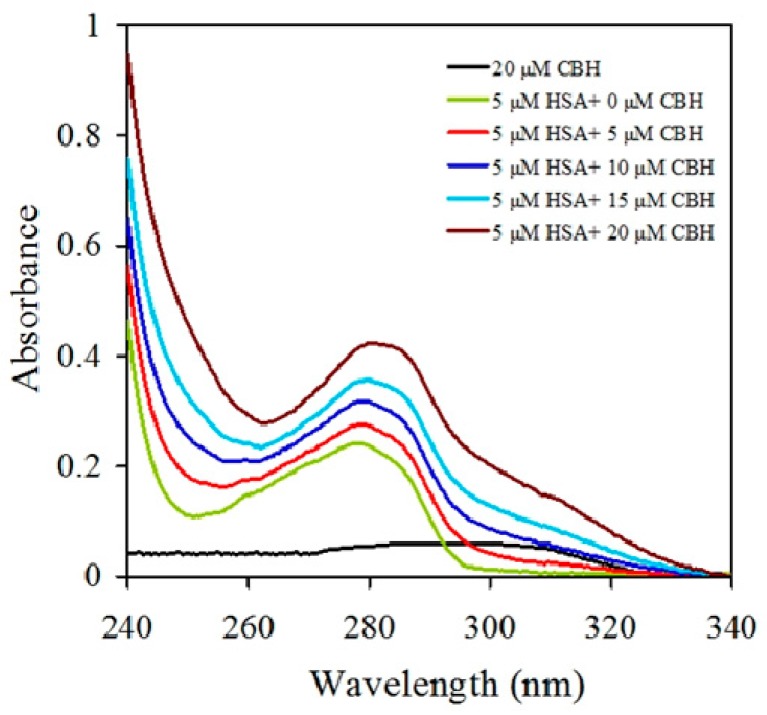
Changes in the UV–VIS absorption spectrum of human serum albumin (HSA) in the absence and presence of different concentrations of cyclobenzaprine hydrochloride (CBH) at pH 7.4 and temperature 25 °C. The black line shows the UV–VIS absorption spectrum of CBH only.

**Figure 3:**
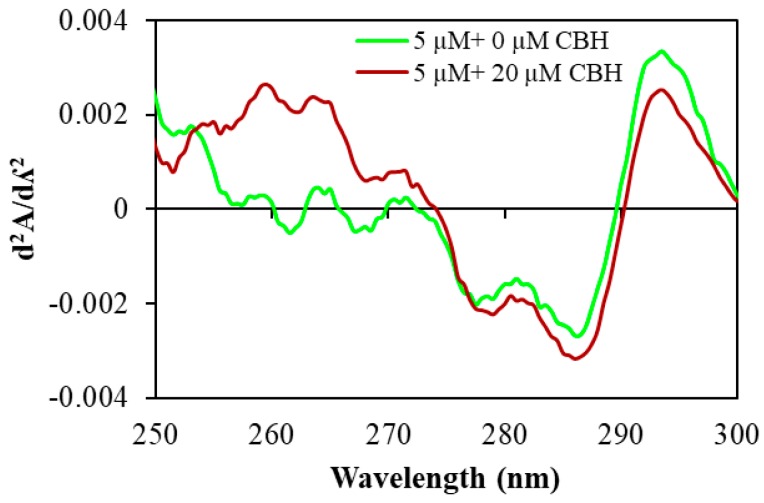
Second derivative absorption spectra of human serum albumin (HSA) in the absence and presence (20 μM) of CBH at pH 7.4 and temperature 25 °C.

UV–VIS absorption was measured to explore CBH-mediated structural changes in HSA. Here we measured UV–VIS absorption spectra of HSA in the absence and presence of CBH at different concentrations (Figure 2). HSA had a major absorption peak at ~280 nm, and this peak is mainly attributed to protein chromophores (Trp and Tyr residues). As seen in Figure 2, upon the addition and subsequent increase in the concentration of CBH, the absorption peak of HSA (between ~270 and 290 nm) showed hyperchromism, i.e., there was a rise in the intensities of HSA absorption. Such changes in the intensities of HSA absorption upon CBH addition indicate that there are micro-environmental alterations around the protein chromophores upon formation of the HSA-CBH complex. Furthermore, the second derivative curves of absorption spectra (Figure 3) of HSA, in the absence and presence of CBH, allowed us to clearly monitor the changes occurring in the micro-environment of the protein aromatic residues Phe, Tyr, and Trp at 260, 270 and 280 nm, respectively.

### 2.2. Alterations in Secondary Structure of HSA

**Figure 4:**
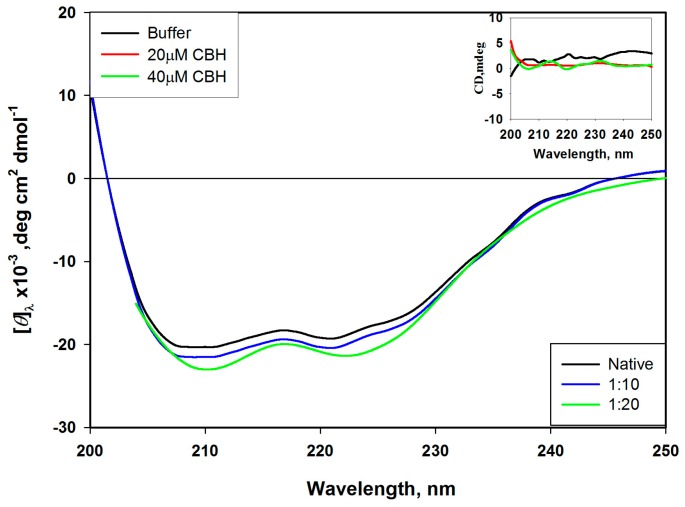
Circular dichroism of the free HSA and HSA–CBH complexes. Circular dichroism (CD) spectra of HSA (2 μM) in the presence of CBH (10 μM, 20 and 40 μM) at pH 7.4. Control spectra of CBH (20 and 40 μM) and buffer are shown in the inset.

We carried out CD experiments to investigate the influence of CBH binding on the structural changes of HSA. The α-helices, β-sheets and β-turns structures are the major secondary structures of proteins and produce specific peaks with unique magnitudes and shapes in the far-UV region [24]. In the far-UV CD spectrum of HSA, the appearance of two negative absorption bands (minima) at 208 nm (π–π*) and 222 nm (n–π*) (Figure 4) characterized the presence of α-helical content in HSA [25]. Figure 4 illustrates the CD spectra changes after binding of CBH with HSA in the far-UV region. Our findings show that the free form of HSA has 54% α-helix and increases to 57%, 58% and 62% after the gradual addition of 10, 20 and 40 μM CBH, respectively. The decrease in the value of MRE_222_ clearly reflects the alterations in the secondary structure of HSA upon CBH binding. The CD spectra for the buffer and CBH (alone) are also shown in the Figure 4 (inset) as a control for comparison with HSA.

### 2.3. Fluorescence Quenching Analysis of HSA in the Presence of CBH

We investigated the interaction of CBH with HSA using fluorescence spectroscopy. This technique was used to understand the quenching mechanism of the HSA-CBH complex. Steady state fluorescence (SSF) spectrometry was used to monitor the change in the micro-environment of Trp within HSA after CBH binding. It has been well established that the aromatic fluorophores of protein moieties (W, F and Y) majorly account for intrinsic fluorescence [26]. These fluorophores are affected by the binding of any ligand to the protein. Trp214 is present in Sudlow’s binding site I (subdomain IIA), and it has been well established that Trp214 has greater fluorescence and a larger quantum yield than other aromatic moieties (Y and P), and is majorly responsible for the intrinsic fluorescence of HSA [10]. The presence of this aromatic residue (Trp214) gave maximum emission at 340 nm in the fluorescence spectrum of HSA [8]. The consistent increase in the concentration of CBH resulted in a decrease in the fluorescence emission of HSA at 340 nm, which clearly indicates that the interaction with CBH quenched the HSA fluorescence emission. This result also suggests that the binding site for CBH was near the aromatic residue (Trp214) of HSA.

**Figure 5:**
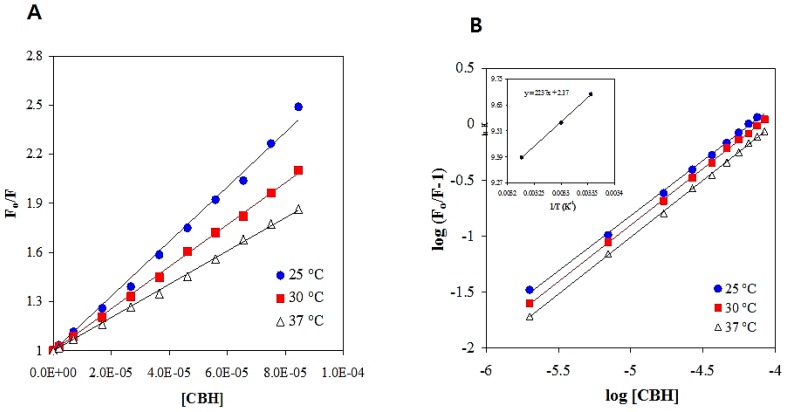
(**A**) Stern-Volmer plot, and (**B**) a double-logarithm plot for the quenching of HSA by CBH at three different temperatures (°C).

Furthermore, we used the Stern-Volmer equation, Equation (6), to analyze the fluorescence quenching data at different temperatures (25, 30, and 37 °C) and reveal the mechanism involved in the quenching of the HSA-CBH interaction. The Stern-Volmer plot constructed for F_0_/F against CBH concentration at three different temperatures is shown in Figure 5A. Here we determined the values of Stern-Volmer constant (KSV) at three different temperatures using linear regression analysis of the Stern-Volmer plots (Table 1). The information provided in Table 1 clearly shows the decrease in the value of KSV and kq with the increase in temperature, which confirms the formation of a ground state complex between HSA and CBH.

### 2.4. Thermodynamic Parameters and Forces Involved in HSA-CBH Binding

In any drug-protein complex, electrostatic interactions, hydrophobic interactions, van der Waals interactions, hydrogen bond formation and steric contacts are the main forces involved in their interaction [27,28]. The intercept and slope of the modified Stern–Volmer plot of log (F_0_/F – 1) vs. log [CBH] were used to calculate the K_b_ and binding stoichiometries (n) for HSA–CBH interaction at various temperatures (as derived from fluorescence quenching experiments) (Figure 5B). The signs and magnitudes of various thermodynamic parameters such as the entropy change (Δ*S*°), free energy change (Δ*G*°), and enthalpy change (Δ*H*°) are vital in any complex formation and can be utilized to identify the different types of forces involved in the formation of HSA-CBH complex [27]. The binding constant (Kb) derived at three different temperatures (25, 30 and 37 °C) were used to determine the Δ*H*° and Δ*S*° values from the slope and intercept values in van’t Hoff Equation (7) (Figure 5B and Table 1). Change in Gibbs free energy (Δ*G*°) for the HSA-CBH complex system at different temperatures was calculated using Equation 8 and the values are shown in Table 1.

The negative value of Δ*G*° at different temperatures suggests that CBH binding with HSA is a spontaneous process. As mentioned above, the value of Δ*H*° and Δ*S*° are important thermodynamic parameters for predicting the involvement of an interacting force(s) in ligand-protein complex formation [27]. A positive value of Δ*S*° is an indication of hydrophobic interactions and other electrostatic forces in the binding of any ligand to protein [27]. Therefore, the positive value of Δ*S*° here is a clear indication that hydrophobic interactions are dominant in the binding of CBH-HSA. A negative value of Δ*H*° indicates both hydrogen bonds and van der Waals are associated with the formation of the ligand-protein complex [29].

### 2.5. Energy Transfer Efficiency and HSA and Binding Distance

**Figure 6:**
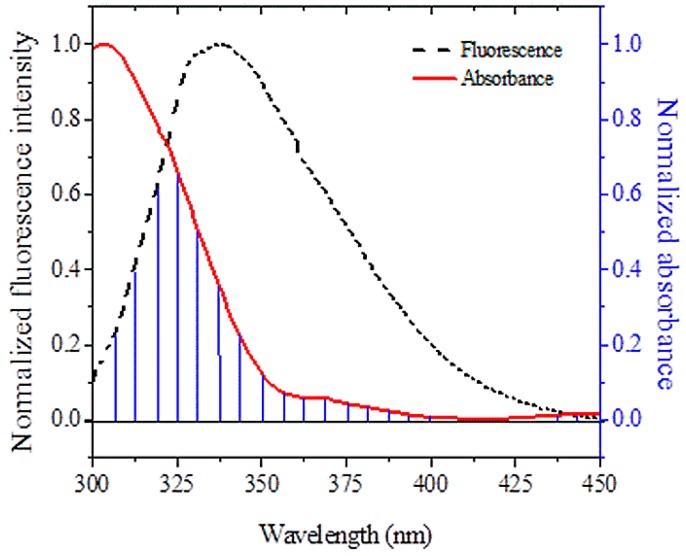
Overlap between the fluorescence emission spectrum of HSA and the absorption spectrum of CBH.

Fluorescence resonance energy transfer was used in this study to measure the distance between the drug (CBH) and the protein (HSA) [30]. Figure 6 shows the overlap of HSA’s fluorescence emission spectra with the UV-absorption spectra of CBH. The process of energy transfer between drug and protein has wide applications and holds great importance in the field of biochemistry [31]. The energy transfer rate depends on the relative orientations of donor and acceptor dipoles, the extent of overlap between the donor’s fluorescence emission spectrum and the acceptor’s absorption spectrum, and the distance (r) between the acceptor and donor. The energy transfer effect is related not only to the distance between the acceptor and donor, but also to the critical energy transfer distance Ro and the efficiency of energy transfer E. We calculated the energy transfer efficiency E between CBH and HSA on the basis of Förster’s energy transfer theory, using the following equation:

(1) $$EFRET=\left(1-\frac{F}{F_{o}}\right)=\frac{R_{o}^{6}}{R_{o}^{6}+r^{6}}$$

where *E*_FRET_ is the efficiency of energy transfer, the fluorescence intensities of HSA in the presence and absence of CBH are denoted by *F* and *F*_0_, respectively, and *r* is the distance between the HSA (donor) and CBH (acceptor). We calculated the value of *R*_o_, which is the critical distance between acceptor and donor when the transfer efficiency is 50% using the following equation:

(2) $$R_{o}^{6}=8.79\times 10^{-25}k^{2}n^{-4}\varphi J$$

where “*K*^2^” is the spatial orientation factor of the acceptor and donor dipole, “*n*” is the refractive index, “*φ*” is the fluorescence quantum yield of the donor, while “*J*” is the degree of overlap between the absorption spectrum of the acceptor and the fluorescence emission spectrum of the donor. *J* was derived using the following equation:

(3) $$J=\frac{\int_{o}^{\infty }F(\lambda )\varepsilon (\lambda )\lambda^{4}d\lambda }{\int_{o}^{\infty }F(\lambda )d\lambda }$$

where *F*(*λ*) is the fluorescence intensity of the donor at wavelength *λ*, while “*ε*(*λ*)” is the molar absorption coefficient for the acceptor at wavelength *λ*. The value of *J* as calculated using Equation (3) was 3.08 × 10^−15^ cm^3^ M^−1^. The values of *R*_o_ and *r* calculated using Equations (1)–(3) were 2.18 nm and 2.34 nm, respectively, while the value of efficiency of energy transfer (EFRET) was 0.39. At 25 °C, the values of *F*_o_ and *F* were 2546 and 1829, respectively. As suggested from the results, the calculated values of Ro and r fell within the scale of 2–8 nm and thereby obeyed the criteria 0.5*R*_o_ < *r* < 1.5*R*_o_ (Table 2 and Figure 6). This indicates that there existed a large probability of energy transfer between the donor and acceptor. Overall these results clearly suggest that CBH acts as a very strong quencher and thereby provides a clue to its localization near the HSA fluorophore.

### 2.6. Denaturation of HSA with Guanidine Hydrochloride (GdmCl)

To determine the effect of CBH binding on the stability of HSA, we measured far-UV CD spectra of HSA in different concentrations of GdmCl in the presence and absence of CBH (1:1 molar ratio) at 25 °C and pH 7.4. From CD spectra, the values of [Θ]_222_ (a secondary structure probe) were determined at different concentrations of GdmCl. The denatured fraction of HSA, *f*_D_ at a given concentration of [GdmCl] was evaluated using the relation,

(4) $$f_{D}=(y-y_{N})/(y_{D}-y_{N})$$

**Figure 7:**
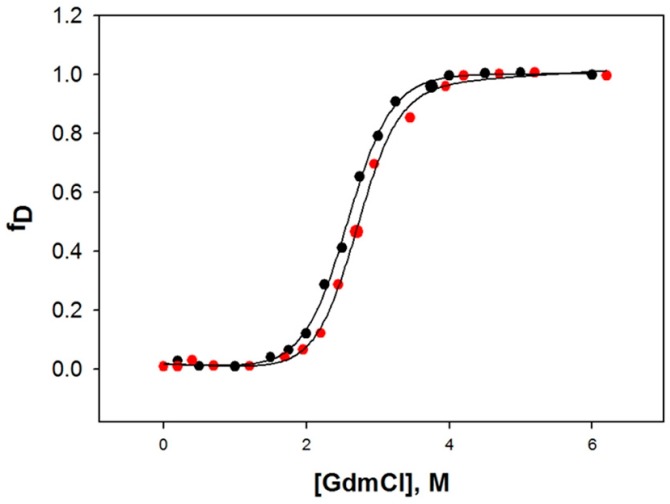
Stability of HSA in presence and absence of cyclobenzaprine. GdmCl-induced denaturation of HSA in the presence and absence of CBH at pH 7.4 and 25 °C.

Over the entire range of denaturant concentration, the unfolding transition of HSA was reversible, both in the absence and presence of CBH. All the analyses were made assuming a two-state model for denaturation, where only the unfolded and native states are significantly populated. A nonlinear least-square method was utilized (Equation (11)) to analyze these plots (*f*_D_ curves), and all the transition curves were analyzed to attain the thermodynamic parameters. Table 3 provides the calculated values of ∆*G*_D_^o^, *m* and *C*_m_. The results obtained from the comparison of *C*_m_ and Δ*G*_D_^o^ values for HSA were in excellent agreement with previous findings [32,33], indicating the accuracy of the data obtained for GdmCl-induced denaturation. The findings show that in the presence of CBH, there is a slight increase in the thermodynamic stability of HSA, i.e., the presence of CBH increases the overall stability of HSA. A higher concentration of GdmCl was required to denature HSA with CBH compared to HSA alone (Figure 7 and Table 3).

### 2.7. Enzyme Activity

**Figure 8:**
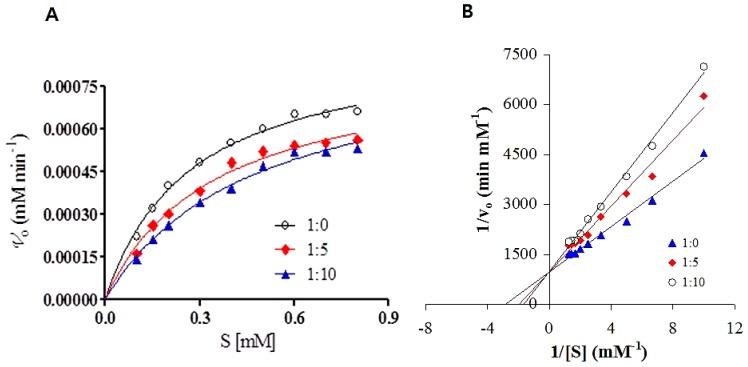
Effect of CBH on the steady-state kinetics of p-NPA hydrolysis by HSA for the hydrolysis of p-NPA by HSA at 25 °C. (**A**) The Michaelis–Menten plot in the absence and presence of CBH. (**B**) Lineweaver–Burk plot for the steady state kinetics of p-NPA hydrolysis by HSA.

Despite being a major transport protein, HSA possesses esterase-like activity to hydrolyze various compounds; one of these is p-NPA [34,35]. R410 and Y411, two reactive residues of subdomain IIIA, are reported to be essential for the esterase-like activity of HSA [8,34,36,37]. K199, H242, and R257 are other major active site residues that are pivotal for the esterase activity of HSA [37,38]. Thus, the esterase-like activity of HSA towards p-NPA was evaluated in the presence and absence of CBH by measuring the formation of p-nitrophenol from p-NPA. The kinetic constants (*K*_m_ and *V*_max_) for the hydrolysis were determined by fitting initial velocity versus (p-NPA) concentration to the Michaelis–Menten equation (Figure 8A). Further, the reciprocal of substrate concentration (p-NPA) initial velocity was plotted as a Lineweaver-Burk plot at increasing CBH molar ratios (1:0, 1:5, and 1:10) to deduce the mechanism involved in the inhibition of the esterase-like activity of HSA by CBH (Figure 8B). Table 4 shows the values for all the kinetic parameters (*k*_cat_ and *K*_m_). Figure 8 illustrates the changes in the esterase-like activity of HSA with increasing concentrations of CBH. The *K*_m_ and *V*_max_ of HSA towards p-NPA were equal to 34.8×10^-2^ mM and 10.2 × 10^−4^ mM min^-1^, respectively; with increasing CBH, the *K*_m_ tended to increase while *V*_max_ remained the same. This increase in *K*_m_ in the presence of CBH and no change in *V*_max_ indicated that CBH exhibits competitive inhibition and binds to the same place that is usually occupied by the substrate. The results obtained in this study accord well with those reported for other myorelaxants, such as tolperisone hydrochloride and eperisone hydrochloride, which are also reported to bind to HSA in a competitive manner [8,10].

### 2.8. Molecular Docking

**Figure 9:**
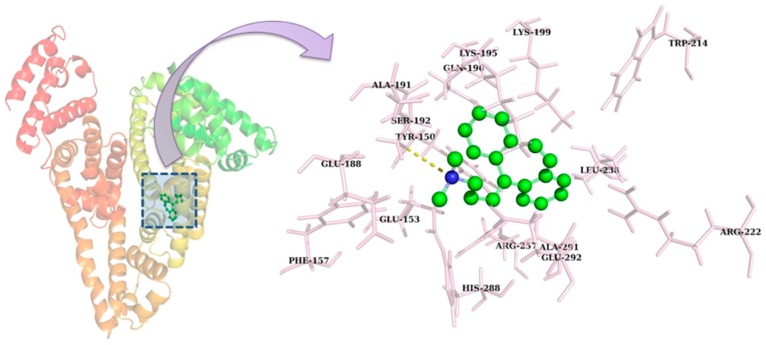
Docked conformation of CBH within the subdomain IIA (Sudlow site I) of HSA and the residues involved in accommodating CBH within its binding site.

Molecular docking studies were performed to explore the interaction of CBH with HSA [39]. Because of its heart-shaped structure, HSA has a central channel that allows the entry and binding of small molecules with its subdomains [40]. The main ligand binding regions of HSA are situated in hydrophobic cavities of subdomains IIA and IIIA, and these subdomains are consistent with Sudlow sites I and II, respectively [41,42]. In this study, an autodock program was used to investigate possible binding conformations of CBH with HSA and the best conformation was selected based on the binding free energy. Table 5 and Figure 9 summarize the binding of CBH within the binding site of HSA, revealing that CBH was accommodated within the cavity of subdomain IIA of HSA. The binding of CBH with HSA was dominated by hydrophobic interactions, and just one hydrogen bond was involved in the binding of CBH to HSA. This *in silico* finding justified the results obtained from the fluorescence quenching thermodynamic parameters, viz., entropy change (Δ*S*) and enthalpy change (Δ*H*). The experimental findings also suggest that the binding forces involved in the binding of CBH within HSA were majorly governed by hydrophobic interactions. Furthermore, these results showed that CBH interacted with HSA with a binding free energy (Δ*G*) of −7.5 kcal mol^−1^, while the fluorescence data showed that CBH fit more favorably in the hydrophobic cavity in subdomain IIA with Δ*G* of −5.78 kcal mol^−1^. Overall, our *in silico* findings are well-justified with regard to our experimental results.

### 2.9. Molecular Dynamics Simulation

**Figure 10:**
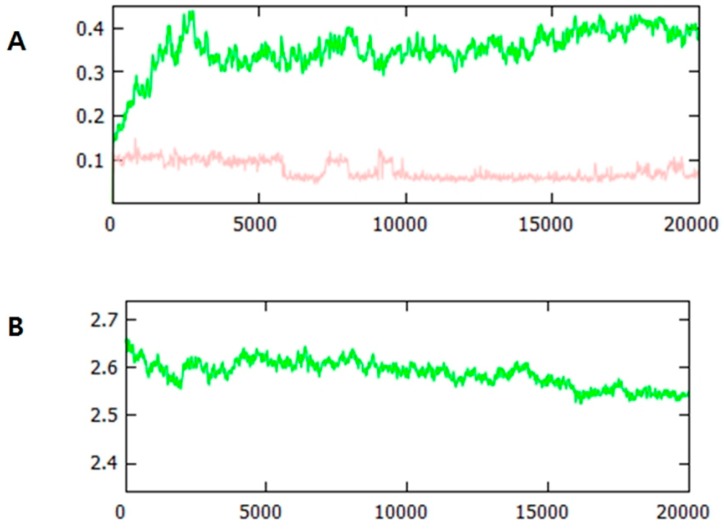
CBH binding mediated changes observed in the structure of HSA during the 20 ns time scale of molecular dynamics simulation. (**A**) RMSD plot of HSA in complex with CBH. HSA in indicated by green line while CBH is indicated by light pink. (**B**) Rg of Cα atoms of HSA in complex with CBH.

Molecular dynamics (MD) were used to further investigate the binding of CBH with HSA and the stability of the complex. MD is a powerful tool for calculating the time-dependent action of a molecular system. Because HSA is an important blood plasma protein, the study of its dynamics in a complex with a ligand holds great importance [43]. Based on the good agreement of our *in silico* and experimental results, we performed the molecular dynamics simulation of HSA in complex with CBH (lowest binding energy docked complex) for a time period of 20 ns. The findings of the MD study allow us to observe the effect of the ligand on the overall structure of HSA. Figure 10 summarizes the CBH binding-mediated changes in the structure of HSA during the 20-ns time scale of the molecular dynamics simulation.

The change in the root mean square deviation (RMSD) values is an indicator of the conformational change within the protein and ligand. The complex remained stable after 5 ns and little fluctuation was observed during the entire time period (Figure 10A). It has been well established that the small changes in the small globular proteins (1–3 Å) are negligible. CBH was stable throughout the simulation, suggesting stable binding to HSA. These predicted RMSD changes within HSA with CBH clearly suggest that the complex was very stable during the entire simulation period. Figure 10B shows the changes in the radius of gyration (Rg) of HSA in the presence of CBH. The Rg indicates the compactness of HSA. In the presence of CBH, HSA initially showed a slightly relaxed conformation, but this tended to decrease after 6 ns. This decrease in the Rg of HSA-CBH implied that the structure became more compact during the simulation time period. This decrease in the Rg of HSA-CBH with time also indicated that the complex was stable.

## 3. Materials and Methods

### 3.1. Reagents

HSA (A1887; globulin and fatty acid free), p-nitrophenyl acetate (N8130) and cyclobenzaprine hydrochloride (MW = 381.85) (BCBJ3705) were purchased from Sigma Aldrich. All reagents used in this study were of analytical grade. The stock solution of HSA (75 μM) was prepared in 20 mM sodium phosphate buffer (pH 7.4). Before sample preparation, the concentration of HSA was estimated spectrophotometrically using $E_{280\;nm}^{1\%}$ = 5.3.

### 3.2. UV-Visible Absorption Spectra Measurements

UV-VIS absorption was measured on a Cary 100 (Varian) spectrophotometer in a 1.0-cm quartz cuvette. UV-VIS spectra of CBH alone and of HSA in the absence and presence of CBH at increasing concentrations were recorded between 240 and 340 nm. All experiments were performed at room temperature, while maintaining HSA at 5 μM.

### 3.3. Fluorescence Quenching and Data Analysis

Fluorescence quenching experiments of HSA in the presence and absence of CBH were performed on a Jasco spectrofluorometer (FP-8300) with an attached water bath. The fluorescence quenching experiments were performed at three different temperatures: 25, 30 and 37 °C. The excitation wavelength was set to 295 nm to avoid Tyr excitation during the quenching experiment [44]. The excitation and emission slit widths were 5 and 2.5 nm, respectively. The emission spectra were recorded at 300–400 nm. To eliminate the inner filter effects of CBH absorbance, excitation and emission wavelengths (295 and 340 nm) were used and corrected using the equation:

(5) $$F_{\mathrm{corrected}}=F_{\mathrm{observed}}\times e^{\frac{\left(\mathrm{Aex}+\mathrm{Aem}\right)}{2}}$$

In this equation, *F*_corrected_ and *F*_observed_ are the corrected and observed intensities of fluorescence, respectively, while *A*_em_ and *A*_ex_ represent the emission and excitation wavelengths of the ligand-protein at 295 and 340 nm, respectively. The Stern-Volmer equation was used to calculate the Trp quenching effect [45]:

(6) $$\frac{F_{o}}{F}=K_{\mathrm{SV}}[Q]+1=k_{q}\tau_{o}[Q]+1$$

where fluorescence emission intensities of HSA without and with quencher (CBH) are denoted by *F*_o_ and *F* respectively. Stern-Volmer quenching and bimolecular quenching rate constants are denoted by *K*_SV_ and *k*_q_, respectively. The molar concentration of CBH (quencher) is denoted by *Q*, while the average excited lifetime of HSA in the absence of quencher is denoted by *τ*_o_ (5.78 × 10^−9^ s).

### 3.4. Thermodynamic Analysis of the Binding Process

The binding constant (*K*_b_) was calculated at different temperatures and further analyzed to understand the nature of CBH binding to HSA. All the thermodynamic data were extracted from the fluorescence quenching assay. During the experimental analysis, it was assumed that there was no significant change in the enthalpy (Δ*H*°) in the studied temperature range.

The van’t Hoff equation was used to calculate the thermodynamics parameters involved in the binding, i.e., entropy change (TΔ*S*°) and enthalpy change (Δ*H*°). The equation used for the calculation was:

(7) $$\ln K=-\frac{\Delta H{}^{\circ}}{RT}+\frac{\Delta S{}^{\circ}}{R}$$

In the above equation, “*K*” is the binding constant at the given temperature, while “*R*” is the ideal gas constant (1.987 cal K-1 mol-1). “*T*” is the absolute temperature in Kelvin. With the previous derived data, we calculated the binding free energy (Δ*G*°) of the HSA-CBH complex using the following equation:

(8) ΔG°=ΔH°−TΔS°

### 3.5. Far-UV Circular Dichroism (CD) Spectropolarimetry for Structural Change Determination

Circular dichroism (CD) measurements were performed at 25 ± 0.2 °C in a 0.1-cm cylindrical cell (Hellma, USA) on a Jasco (J-815) spectropolarimeter. The presented spectra were baseline corrected, and final values of the CD spectra were selected as the average of four repetitions obtained at a scan rate of 50 nm min^–1^ from 200 to 250 nm. For the baseline correction, the buffer solution used in this study was measured in the same way, but no HSA was added. All the CD scans were performed under identical conditions, and the baseline (corrected) was subtracted automatically from the HSA spectra. The mean residue ellipticities (MRE) of the native and the CBH-complex form of HSA were calculated using:

(9) $$MRE=\frac{\Theta_{obs}(m{}^{\circ})}{10\times n\times C\times l}$$

In Equation (9), Θ*obs* is the observed ellipticity in m°, *C* is the HSA concentration (2 μM), *n* is the number of peptide bonds in the protein chain (*n* = 585–1), and the path length of the cuvette is *l*. The MRE values were used to calculate the total α-helical contents of HSA using the equation derived by Chen et al. [46].

(10) $$\%\alpha helix=\left(\frac{MRE_{222nm}-2340}{30300}\right)\times 100$$

### 3.6. Guanidine Hydrochloride-Induced Unfolding Studies in the Absence and Presence of CBH

Guanidine hydrochloride (GdmCl)-induced unfolding of HSA was measured in the presence and absence of CBH (at 25 °C and pH 7.0) at MRE 222 nm on a Jasco (J-815) spectropolarimeter. A MRE of 222 nm (deg cm^2^ dmol^−1^) was derived from the raw data generated by CD using Equation (9).

The unfolding transition curve of HSA was reversible in the entire [g] range (molar concentrations of GdmCl) in the presence of CBH. The non-linear least-squares method was used to analyze the entire data set (*y*(g), [g]) of the denaturant-induced transition curve using the following equation [47]:

(11) $$y(g)=\frac{y_{N}(g)+y_{D}(g)\times e^{\left[-(\Delta G_{D}^{o}+m_{g}[g])/RT]\right]}}{1+e^{\left[-(\Delta G_{D}^{o}+m_{g}[g])/RT)\right]}}$$

where the optical properties (observed) at [g] are denoted by“*y*(g)”. The optical properties of the native and unfolded HSA are denoted by “*y*_N_” and “*y*_D_”, respectively. The values of “*y*_N_” and “*y*_D_” were measured under the same experimental conditions as “*y*(g)”. Change in the Gibbs energy (in the absence of GdmCl) is denoted by ∆*G*_D_^o^, *m*_g_ is the slope (∂Δ*G*_D_/∂[g])_T,P_, and “*R*” and “*T*” are the universal gas constant and temperature, respectively. During the analysis of the GdmCl-induced transition curve, it was assumed that the unfolding of HSA was a two-state process.

### 3.7. Esterase-Like Activity of HSA

The esterase-like activity of HSA was measured to determine the effect of CBH binding on the functionality of HSA. Steady state kinetics at pH 7.4 and 25 °C were studied to evaluate the effect of CBH binding on the catalytic activity of HSA toward p-nitrophenyl acetate (p-NPA). The concentration of HSA was fixed at 8 µM while the concentration of p-NPA varied from 0 to 0.8 mM. The reactions were incubated at 25 °C for 12 h before carrying out activity measurements at different molar ratios of HSA:CBH (1:0, 1:5, and 1:10). The rate of p-nitrophenol hydrolysis was determined by monitoring the appearance of p-NPA at 405 nm.

### 3.8. Molecular Docking

A molecular docking study was performed to gain better insight into the binding of CBH within the active site of HSA. AutoDock 4.2 [48] was used to perform molecular docking experiments. First, the known three-dimensional structure of HSA was retrieved from the RCSB Protein Data Bank [49] (PDB id: 1AO6), while the 3-D structure of CBH was retrieved from the PubChem Database (pubchem id: 22576). The structure of HSA was prepared for molecular docking by removing all the water molecules and HETATM. The molecular docking of CBH against HSA was then performed for 20 runs using the Lamarckian genetic algorithm with default parameters. Out of 20 different conformers, the best one was selected based on the binding free energy.

### 3.9. Molecular Dynamics

The docked complex of HSA-CBH was used as a starting point for the molecular dynamics (MD) study. This complex was subjected to the MD study to better understand the binding of CBH with HSA. The GROMACS 4.6.7 package with gromos96 force field was used to perform the MD simulations [50,51,52]. The explicit SPC water model was used to solvate the HSA-CBH complex within a dodecahedron box, with a margin of 0.1 nm between the solute and the box walls [53]. The system charge was neutralized by the appropriate addition of sodium and chloride counter ions to bring the concentration to 0.1 M. To calculate the long-range electrostatic interactions, the particle-mesh Ewald method [54] was used with a cutoff distance of 0.1 nm. The van der Waals interactions were evaluated using the Lennard-Jones 6–12 potential with a cutoff distance of 0.1 nm. The LINCS algorithm of fourth order expansion was used to constrain bond lengths [55]. After the solvation and neutralization of the complex system, steric clashes between atoms were removed by subjecting the complex to energy minimization for 10,000 steps using the steepest-descent method. The system was equilibrated for 1 ns with position restraints on all heavy atoms. Berendsen weak coupling schemes [56] were used to maintain the system at 300 K and 1 atm using separate baths for the solute and the solvent. Initial velocities were generated randomly using a Maxwell Boltzmann distribution corresponding to 300 K. Neighbor lists were updated every 10 fs using a group cut-off scheme. Finally, the production run was performed for 20 ns without restraints at 300 K in the isothermal-isobaric (NPT) ensemble. Furthermore, xmgrace (http://plasmagate.weizmann.ac.il) was used to prepare graphs; VMD [57] and PyMol (The PyMOL Molecular Graphics System, Version 1.7 Schrödinger, LLC.) packages were applied for system inspection and further analysis.

## 4. Conclusions

This is the first study demonstrating the detailed binding mechanism of CBH on HSA, along with its influence on stability, catalytic activity, and conformation changes within HSA. HSA is one of the most ideal proteins for investigating the interactions of various drugs, chemicals etc. The addition of CBH resulted in hyperchromism in the HSA absorption peak (between ~270 and 290 nm). The results obtained from CD showed that the binding of CBH leads to increased α-helical content of HSA. The fluorescence quenching experiments indicated that the quenching of HSA by CBH is a result of the static quenching mechanism. As revealed from the molecular docking results, similar to other muscle relaxant drugs, CBH binds within subdomain IIA (Sudlow site I) of HSA. In our previous study on quenching of HSA by other muscle relaxant drugs, the binding free energy (Δ*G*°) was −5.93 kcal mol^–1^ and −6.8 kcal mol^−1^ for tolperisone hydrochloride and eperisone hydrochloride, respectively. In this study, the calculated binding free energy of CBH was −5.75 kcal mol^−1^, which indicates strong binding of this drug to HSA. In summary, this study provides valuable insight into the binding mechanism of CBH to HSA, which is very helpful in improving our understanding of the effect of muscle relaxant drugs on skeletal muscle during their transportation and distribution in the blood. This study not only provides an overview of the drug-induced micro-environmental changes in HSA, but also gives accurate and valuable insights into the binding of CBH to HSA. This information will be useful in the development of better muscle relaxant drug candidates and for improving drug delivery.

## Figures and Tables

**Table 1:** The quenching constant (K_sv_), binding constant (K_b_), binding stoichiometry (*n*), and thermodynamic parameters between HSA and CBH from fluorescence quenching experiments at 25, 30 and 37 °C.

Parameter	25 °C	30 °C	37 °C
*n* (binding stoichiometry, HSA:CBH)	0.99 ± 0.11	1.01 ± 0.13	1.101 ± 0.12
K_SV_ (Stern-Volmer constant, M^−1^)	1.7 ± 0.21 × 10^4^	1.3 ± 0.24 × 10^4^	1.0 ± 0.31 × 10^4^
K_b_ (binding constant, M^−1^)	1.6 ± 0.22 × 10^4^	1.4 ± 0.19 × 10^4^	1.2 ± 0.23 × 10^4^
k_q_ (bimolecular quenching rate constant, M^−1^ s^−1^)	2.6 ± 0.12×10^12^	2.2 ± 0.15 × 10^12^	1.7 ± 0.19 × 10^12^
Δ*H*° (binding enthalpy, kcal mol^−1^)	-	−4.44±0.16	-
TΔ*S*° (entropy change, kcal mol^-1^)	1.28 ± 0.18	1.30 ± 0.15	1.33 ± 0.16
Δ*G*° (Gibbs free energy change, kcal mol^-1^)	−5.72 ± 0.12	−5.75 ± 0.15	−5.78 ± 0.14

The data are the means ± standard deviations of three independent trials.

**Table 2:** FRET from steady state measurements performed at 25 °C.

Parameter	Value
J (cm^−3^ M^−1^)	3.08 × 10^−15^
Ro (nm)	2.18
*r* (nm)	2.34
EFRET	0.39
F_o_	2546
F	1829

**Table 3:** Thermodynamic parameters associated with GdmCl-induced denaturation of HSA in the presence and absence of CBH.

Protein	Δ*G*_D_^o^, kcal mol^−1^	*m*_g_, kcal mol^−1^ M^−1^	*C*_m_, M
0.0 μM CBH	4.92 ± 0.24	1.98 ± 0.14	2.48
100 μM CBH	5.42 ± 0.19	2.01 ± 0.18	2.69

**Table 4:** Kinetic parameters describing Michaelis-Menten constant, *V*_max_, catalytic constant and catalytic efficiency of HSA in the absence and presence of increasing CBH concentrations.

HSA:CBH	*V*_max_ (mM min^−1^)	*K*_m_ (mM)	*k*_cat_ (min^−1^)	*k*_cat_/*K*_m_ (mM^−1^ min^−1^)
1:0	10.2 × 10^−4^	34.8 × 10^−2^	12.8 × 10^−2^	0.36
1:5	10.2 × 10^−4^	50.6 × 10^−2^	12.8 × 10^−2^	0.25
1:10	10.0 × 10^−4^	60.0 × 10^−2^	12.5 × 10^−2^	0.20

All measurements were carried out in 20 mM sodium phosphate buffer pH 7.4 at 25 °C. Values of *V*_max_ and *K*_m_ were derived from the Lineweaver-Burk equation. *k*_cat_/*K*_m_; catalytic efficiency. *K*_cat_; catalytic constant (*V*_max_ = *k*_cat_ × enzyme concentration). The concentration of HSA was 8 μM.

**Table 5:** Binding of CBH within the active site of HSA and the residues involved in binding.

Complex	Binding Free Energy (Δ*G*) (kcal mol^−1^)	Residues Involved	
		Hydrogen Bond	Hydrophobic Interaction
HSA-CBH	−7.55	S192	Y150, E153, S192, K195, Q196, K199, L238, R257, H288, A291, E292

## Abbreviations

CD	Circular dichroism
CBH	Cyclobenzaprine hydrochloride
∆*H*	Enthalpy
HSA	Human serum albumin
*K*_m_	Michaelis-Menten constant
λmax	Wavelength maxima
MRE	mean residue ellipticity
*V*_max_	maximum velocity
